# Pathways between probation and addiction treatment in England

**DOI:** 10.23889/ijpds.v8i2.2321

**Published:** 2023-09-14

**Authors:** Brian Eastwood, Patrick Horgan, Michael Kettle, Grant Henderson, Simon Corr, Nino Maddalena, Guy March, Mariana Bazely, James Osmond, Matthew Katz, Vincent Lam, Thomas Morris, Rosie Chalam-Judge, Eleanor Martin, Lisa Robinson

**Affiliations:** 1 Department of Health and Social Care, London, United Kingdom; 2 Ministry of Justice, London, United Kingdom

## Objectives

The Criminal Justice Act (2003) introduced Alcohol Treatment Requirements (ATR) and Drug Rehabilitation Requirements (DRR). The study’s objective was to better understand the pathways between ATRs/DRRs and specialist addiction treatment services.

## Methods

The Ministry of Justice's Splink software was used to probabilistically link individuals present in MoJ's nDelius probation data with those in structured substance misuse treatment in the community from the National Drug Treatment Monitoring System (NDTMS), for individuals in England sentenced between 2018 and 2022. The sociodemographic characteristics and clinical profiles of the group were explored, as well as their treatment outcomes. Mixed-effects logistic regressions were used to investigate what factors influence positive outcomes from ATRs/DRRs.

## Results

Overall, 38.9% of offenders on ATRs/DRRs were either engaged with treatment services on their sentence date or engaged with treatment services after being sentenced. Offenders with an ATR appeared to engage more (45.9%) than those with a DRR (33.1%).

At three weeks after the sentence date, 26% of offenders with an ATR were identified in treatment, compared to 20% of offenders with a DRR. Of the 15,121 offenders engaged in treatment, 27% remained on the same initial treatment journey until the end of the observation period. 37% dropped out of treatment, while 35% successfully completed this treatment journey (i.e., recovered from their substance use). 1.4% died. Results were also produced on factors were associated with making it into treatment, and with successful treatment outcomes.

## Conclusion

For the first time in England, addiction treatment data and probation data have been linked to produce a more accurate picture of the journey of individuals sentenced to ATRs/DRRs. The findings suggest more work is required to optimise the pathways between probation and specialist addiction treatment services.

